# Nutritional and immunological parameters as prognostic factors in patients with advanced oral cancer^☆^

**DOI:** 10.1016/j.bjorl.2021.11.003

**Published:** 2021-12-04

**Authors:** Lorenzo Fernandes Moça Trevisani, Isabelle Fernandes Kulcsar, Ana Kober Nogueira Leite, Marco Aurélio Vamondes Kulcsar, Graziele Aparecida Simões Lima, Rogerio Aparecido Dedivitis, Luiz Paulo Kowalski, Leandro Luongo Matos

**Affiliations:** a Universidade de São Paulo, Faculdade de Medicina, Programa de Pós-Graduação em Anestesiologia, Ciências Cirúrgicas e Medicina Perioperatória, São Paulo, SP, Brazil; b Universidade Anhembi Morumbi, São Paulo, SP, Brazil; c Universidade de São Paulo, Faculdade de Medicina, Instituto do Câncer do Estado de São Paulo (Icesp), Departamento de Cirurgia de Cabeça e Pescoço, São Paulo, SP, Brazil; d Universidade de São Paulo, Faculdade de Medicina, Instituto do Câncer do Estado de São Paulo (Icesp), São Paulo, SP, Brazil; e Universidade de São Paulo, Faculdade de Medicina, Hospital das Clínicas, Departamento de Cirurgia de Cabeça e Pescoço, São Paulo, SP, Brazil; f Faculdade Israelita de Ciências da Saúde Albert Einstein, São Paulo, SP, Brazil

**Keywords:** Oral cavity, Carcinoma, squamous cell, Body weight changes, Nutrition, Prognosis

## Abstract

**Objective:**

The aim of the present study was to analyze the prognostic relationship of weight loss and preoperative hematological indexes in patients surgically treated for pT4a squamous cell carcinoma of the oral cavity.

**Methods:**

A retrospective cohort study.

**Results:**

Percent weight loss greater than 10% was identified in 49 patients (28.2%), and any weight loss in relation to the usual weight occurred in 140 patients (78.7%). Percent weight loss greater than 10% (HR = 1.679), Red cell distribution width (RDW) values greater than 14.3% (HR = 2.210) and extracapsular spread (HR = 1.677) were independent variables associated with risk of death.

**Conclusion:**

Patients with advanced squamous cell carcinoma of the oral cavity present significant weight loss and as significantly immunocompromised. Increased values of RDW and higher percentages of weight loss in relation to the individual's usual weight, together with extracapsular spread of metastatic lymph nodes, were risk factors for lower survival, regardless of other clinical and anatomopathological characteristics.

**Level of evidence:**

3.

## Highlights

• Patients with oral SCC present significant weight loss and immune compromise.
• Increased values of RDW and high weight loss were risk factors for lower survival.
• Patients undergoing treatment must receive a complete nutritional evaluation.
• Nutritional intervention can be effective, preventing nutritional deterioration.

## Introduction

Oral cavity cancer is a highly prevalent disease worldwide.^1^, ^2^ In 2020 in Brazil, according to the estimates of the Brazilian National Cancer Institute (INCA), approximately 11,000 new cases of the disease are expected for men, with Oral Squamous Cell Carcinoma (OSCC) being the main histological type.^3^, ^4^, ^5^

Malignant tumors are known to cause chronic inflammation and malnutrition,^6^ however, due to anatomical location and symptoms, patients who present with OSCC have a higher propensity to weight loss. This weight loss is involuntary and can affect 31%–87% of patients. In more advanced stages, with difficulties in chewing and swallowing, there is a significant worsening in nutritional status.^7^

As the majority of cases in the Brazilian population are diagnosed at advanced clinical stages,^8^ this population presents with severe impairment of body mass^9^ and nutritional status has been previously associated with mortality in cancers from different sites, including head and neck.^10^, ^11^, ^12^ The immune system is also affected by the body composition of each individual.^13^

Hematological inflammatory markers, such as neutrophil/lymphocyte ratio, have been associated with higher mortality in oncological patients and, therefore, have been described as predictors of prognosis in different neoplasms.^14^, ^15^, ^16^ Red cell Distribution Width (RDW) reflects impaired erythropoiesis and abnormal red blood cell survival, while the heterogeneity of red blood cell size correlates with inflammation and undernutrition status.^17^, ^18^ Recent studies have also shown that RDW could be a prognostic factor in several carcinomas.^19^, ^20^

Thus, nutritional assessment associated with other prognostic factors, such as the state of the immune system, can be of great importance for the indication of supportive care.^21^ Malnourished patients have even less tolerance for and response to antineoplastic treatment, resulting in treatment delays, reduced immunological competence, increased postoperative complications, and, consequently, lower survival expectancies.^22^

The present study aimed to evaluate the influence of nutritional, immunological and inflammatory factors on mortality in patients undergoing upfront curative surgical treatment for advanced oral squamous cell carcinoma, patients more suitable to nutritional and immunological disorders, for whom these additional comorbidities could contribute even more with worse prognosis. Moreover, well known prognostic factors were also analyzed.

## Methods

This was a retrospective study approved by the Institutional Research Ethics Committee (protocol number 228/14; CAAE: 32884214.5.0000.0065).

Patients over 18 years old who were consecutively surgically treated with curative intent for advanced OSCC (stage pT4a according to the 8thedition of AJCC manual^23^: those with moderately advanced local disease with the invasion of adjacent structures of the oral cavity such as cortical bone of the mandibule or maxilla, maxillary sinus or skin of the face) at our Institution from 2010 to 2017 were included to have a minimum of 3 years of follow-up. Patients who had not been evaluated by the hospital's nutrition team or had a history of previous cancer treatment for any other neoplasm or had distant metastasis at the diagnosis of the OSCC were excluded.

Demographic and epidemiological data were collected retrospectively through consultation of electronic medical records. All patients were followed monthly in the first and bimonthly in the second year after surgery and twice a year after the third year of follow-up.

The data collected by the nutrition team during outpatient and hospital evaluations were used in the study and served as a basis for calculating several parameters. Usual weight was established as the one stated by the patient and/or family member in the first consultation; the period before the disease referred to when the patient was considered healthy and performing usual daily life activities. The objective measurement of weight and height were obtained from the medical records. Then, the calculation of body loss was performed in relation to the usual weight expressed as a percentage [% = ((Normal weight - current weight) – 100/usual weight)]. Body Mass Index (BMI) was calculated using the formula: (BMI = P [weight in kilos]/A² [height × height, in meters]) and classified as either underweight adults (<18.50 kg/m^2^), low weight Grade 1 (17–18.49 kg/m^2^), low weight Grade 2 (16.00–16.99 kg/m^2^), low weight Grade 3 (<16 kg/m^2^), eutrophic (18.5–24.99 kg/m^2^), preobesity (25–29.99 kg/m^2^), class I obesity (30–34.99 kg/m^2^), class obesity II (35–39.99 kg/m^2^), or obesity class III (>40 kg/m^2^) according to the World Health Organization recommendation.^3^ For the elderly (60+ years-old), they were classified as underweight (<23 kg/m^2^), normal weight (23–27.9 kg/m^2^), overweight (28–29.9 kg/m^2^) or obese (>30 kg/m^2^) according to *Organización Panamericana de la Salud* (OPAS), 2002.^24^ Other well-known nutritional parameter were also calculated for each patient, such as the ideal weight (mean BMI × height^2^) and the minimum healthy weight (minimum reference value of the normal weight by BMI × height^2^ for each individual), thus classifying the number of patients who were below the ideal weight and by how much.

To assess immunological status, data was collected from the blood tests performed in the preoperative evaluation, such as erythrocytes, Hemoglobin (Hb), Hematocrit (Ht), Mean Corpuscular Volume (MCV), Mean Corpuscular Hemoglobin (MCH), Mean Corpuscular Hemoglobin Concentration (MCHC), Red cell Distribution Width (RDW), leukocytes, neutrophils, eosinophils, basophils, lymphocytes, monocytes, platelets, Mean Platelet Volume (MPV), neutrophil/lymphocyte ratio.

For Overall Survival (OS), the follow-up time was calculated from the date of surgery until the date of death or the date of the last medical appointment for living patients.

The values of continuous variables were described using the means and Standard Deviation (SD). Relative and absolute frequencies were used to describe qualitative data. The cut-off values for quantitative variables were determined by ROC (Receiver Operating Characteristics) curve analysis and by clinical criteria. Cox's regression method was used in univariate analyses and as a multivariate model, estimating the Hazard Ratio (HR) values and the respective 95% Confidence Intervals (95% CI). Variables with *p*-value <0.10 on univariate analysis were selected for multivariate analysis, except in situations of codependency. The Kaplan-Meier method was used in the survival analyses, and the log-rank test was applied to compare the curves. The statistical program SPSS® version 26.0 (SPSS® Inc; Illinois, USA) was used for all statistical analyses. A *p*-value equal to or less than 5% (*p* ≤ 0.05) was adopted as a level of statistical significance.

## Results

In total, 178 patients surgically treated for pT4a stage OSCC were evaluated. The patients were predominantly male (77.5%), between the fifth and seventh decades of life, with a high prevalence of smokers (83.1%) and alcoholics (70.2%), and with the most prevalent primary floor of the mouth (41.6%). In addition, most patients underwent R0 resections (83.1%), with neoplasms showing perineural invasion (67.4%) and lymph node metastases (62.8%), mostly with extracapsular spread (61.7%). Overall, 128 patients underwent adjuvant radiotherapy (72.3%), and 56 patients (31.8%) also received adjuvant chemotherapy. Locoregional recurrence was observed in 46 patients (26%) and distant metastases in 30 (16.9%) (Table 1).

**Table 1:** Demographic, anatomopathological, treatment and outcome data of the patients with locally advanced (pT4a) oral cancer included in the study.

Features	Results
Demographic data	
Male	138 (77.5%)
Female	40 (22.5%)
Age (mean ± SD)	59.5 ± 11.5 years
Primary site	
Retromolar area	31 (17.4%)
Lower alveolar ridge	22 (12.4%)
Upper alveolar ridge	11 (6.2%)
Tongue	17 (9.6%)
Buccal mucosa	13 (7.3%)
Hard palate	10 (5.6%)
Floor of the mouth	74 (41.6%)
Smokers	148 (83.1%)
Alcohol abuse	125 (70.2%)
Anatomopathological data	
Free resection margins	148 (83.1%)
Degree of differentiation	
Well	33 (18.8%)
Moderately	125 (71.0%)
Poor	18(10.2%)
Perineural invasion	120 (67.4%)
Angiolymphatic invasion	62 (34.8%)
Depth of invasion (mean ± SD)	2.5 ± 1.4 cm
pN (pathological lymph-nodes status)	
pN0	63 (37.2%)
pN1	14 (8.2%)
pN2a	9 (5.3%)
pN2b	16 (9.4%)
pN2c	9 (5.3%)
pN3b	59 (34.7%)
Extracapsular spread	66 (61.7%)
Treatment/clinical outcome	
Adjuvant radiation therapy	128 (72.3%)
Adjuvant chemotherapy	56 (31.8%)
Locoregional recurrence	46 (26%)
Distant metastasis	30 (16.9%)
Death	101 (56.7%)

The nutritional and immunological data of the patients included are shown in Table 2. A percent loss of usual weight greater than 10% was identified in 49 patients (28.2%), and any weight loss in relation to the usual weight occurred in 140 patients (78.7%). Altogether, 171 patients (96.1%) used enteral nutritional therapy, 43 patients (39.8%) after one year of surgery were still on the exclusive enteral diet and 151 (84.8%) patients were followed up with a nutrition team. The mean usual weight was 67.4 kg, the mean percentage of loss in relation to the usual weight was 6.4%, the mean BMI was 23 kg/m^2^, the mean time of use of enteral nutritional therapy was 5 months and the mean number of consultation sessions with a nutritionist in the first year was seven.

**Table 2:** Hematological and nutritional data of the patients included in the study.

Features	Result
**Quantitative data**^a^	
Hemoglobin (Hb)	12.3 ± 2.2 g/dL
Hematocrit (Ht)	42.7 ± 47.0%
Erythrocytes	4.0 ± 0.7 millions/mm^3^
RDW	14.0 ± 1.5%
Leukocytes	15.8 ± 69.8^a^1000/mm^3^
Neutrophils	8.0 ± 5.7^a^1000/mm^3^
Lymphocytes	12.6 ± 156.3^a^1000/mm^3^
Neutrophil/lymphocyte ratio	5.7 ± 5.4
Platelets	269.3 ± 102.06^a^1000/mm^3^
Usual weight	67.4 ± 15.3 kg
Weight on the day before surgery	62.7 ± 14,6 kg
Weight loss over usual weight	6.4 ± 7.7%
Body Mass Index (BMI)	23.0 ± 4.6 kg/m^2^
Minimum healthy weight	53.9 ± 6.5 kg
Ideal weight according to mean BMI	62.2 ± 6.5 kg
Time of use of feeding tube (postoperative period)	5.2 ± 4.2 months
Time of oral supplement use (postoperative period)	1.9 ± 3.0 months
Number of consultations with nutritionists in the first year	7.0 ± 4.9
**Stratified data**	
Hb < 14.3 g/dL	143 (80.3%)
Hb < 10 g/dL	30 (16.9%)
RDW > 14.3%	54 (30.3%)
Neutrophil/leukocyte ratio > 2.2	144 (80.9%)
Weight loss over usual weight > 10%	49 (28.2%)
Weight loss in relation to usual weight (yes)	140 (78.7%)
Malnourished/underweight (parameter)	39 (22.0%)
Under healthy minimum weight (parameter)	37 (20.8%)
Use of feeding tube in the postoperative period	171 (96.1%)
Use of oral supplement in the postoperative period	86 (48.3%)
Postoperative nutritional follow-up	151 (84.8%)
≥3 consultations	139 (78.1%)
≥4 consultations	129 (72.5%)
≥6 consultations	111 (62.4%)
≥10 consultations	57 (32.0%)
Diet in the first postoperative year	
Exclusive oral	60 (55.6%)
Exclusive Enteral	43 (39.8%)
Mixed	5 (4.6%)

a Mean ± Standard Deviation.

**Figure 1:**
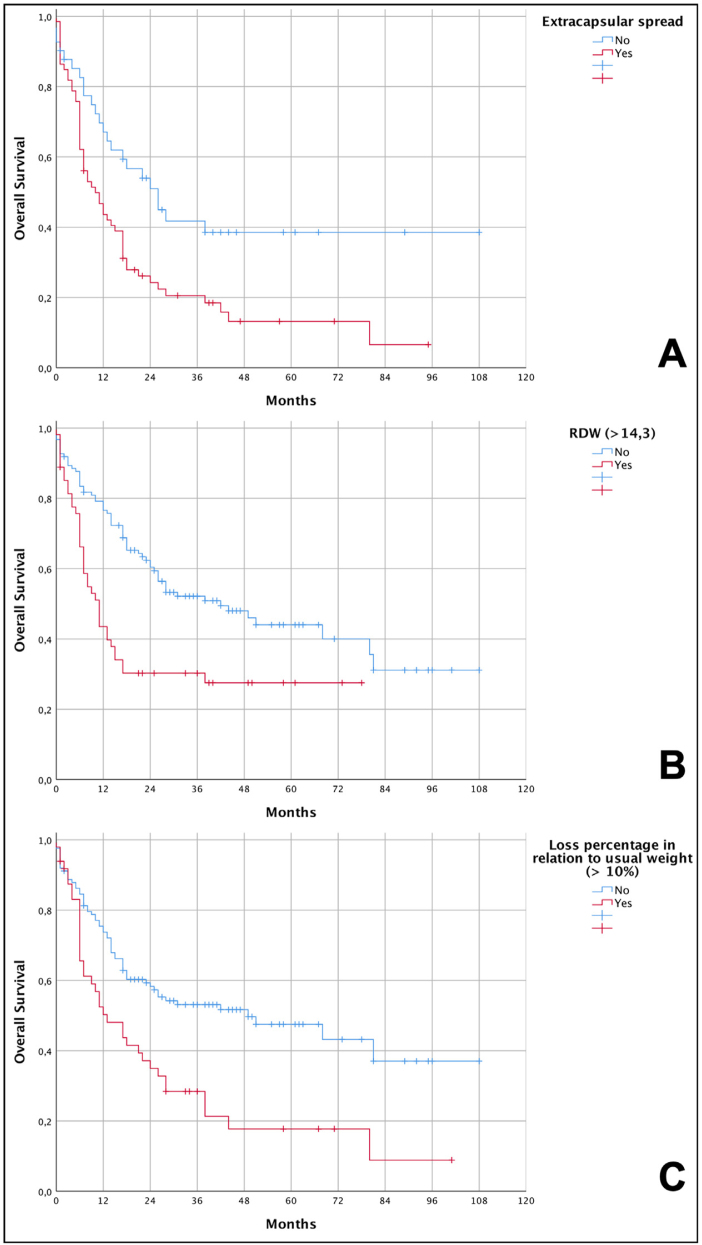
Kaplan-Meier curves showing the overall survival of patients with advanced squamous cell carcinoma (pT4a) of the oral cavity in relation to the independent risk variables. (A) Lower overall survival in patients with extracapsular spread (6.6% vs. 38.5%; *p* = 0.004 – log-rank test); (B) lower overall survival in patients with RDW < 14.3% (27.5% vs. 31.1%; *p* < 0.001 – log-rank test); (C) lower overall survival in patients with weight loss greater than 10% in relation to their usual weight (8.9% vs. 37%; *p* < 0.001 – log-rank test).

Univariate analysis (Table 3 and Fig. 1) showed that perineural invasion, angiolymphatic invasion, presence of lymph node metastases, extracapsular spread of lymph node metastases, hemoglobin levels below 10 g/dL, RDW above 14.3% and weight loss percentage above 10% of the usual weight were associated with lower survival rates.

**Table 3:** Univariate analysis of factors related to the death of patients with advanced squamous cell carcinoma of the oral cavity (pT4a).

Features	HR	95% IC	*p*
Age	1.012	0.994–1.030	0.190
Sex	0.911	0.563–1.474	0.704
Smoking	0.921	0.546–1.553	0.758
Alcohol abuse	0.830	0.545–1.265	0.386
Positive margins	1.196	0.725–1.973	0.483
Poorly differentiated	1.464	0.779–2.752	0.237
Perineural invasion	1.865	1.170–2.975	0.009
Angiolymphatic invasion	1.938	1.301–2.888	0.001
Depth of invasion > 10 mm	1.808	0.984–3.320	0.056
Lymph node metastases	3.406	2.113–5.492	<0.001
Extracapsular spread	2.001	1.225–3.268	0.006
Time to adjuvant radiotherapy (>4 months)	1.136	0.629–2.052	0.673
Hemoglobin < 14.3	1.090	0.672–1.766	0.728
Hb < 10 g/dL	1.805	1.100–2.962	0.019
RDW > 14.3%	2.042	1.355–3.077	0.001
Neutrophil/lymphocyte ratio > 2.2	1.706	0.984–2.956	0.057
Percentage of loss compared to usual weight > 10%	2.040	1.351–3.079	0.001
Weight loss in relation to usual weight (yes)	1.271	0.743–2.174	0.381
Malnourished/Underweight	1.096	0.682–1.759	0.706
Under healthy minimum weight (yes)	1.112	0.687–1.800	0.667
Below ideal weight according to mean BMI (yes)	1.218	0.813–1.825	0.339
Use of enteral tube	0.560	0.244–1.282	0.170
Use of oral supplement	0.919	0.621–1.361	0.675
Follow-up with nutrition team after surgery	0.812	0.475–1.388	0.448

HR, Hazard Ratio; 95% CI, Confidence Interval 95%; *p*, value of *p*.

Multivariate analysis using the Cox regression model (Table 4) showed that percent weight loss greater than 10% (HR = 1.679, 95% CI 1.046–2.693, *p* = 0.032), RDW values greater than 14.3% (HR = 2.210, 95% CI 1.378–3.551, *p* = 0.001) and extracapsular spread (HR = 1.697, 95% CI 1.018–2.831, *p* = 0,043) were independent variables associated with the risk of death. The presence of lymph node metastases itself was not included in multivariate analysis because the great majority of these patients had also extracapsular spread, denoting intrinsic dependence of both variables.

**Table 4:** Multivariate analysis of risk factors for death of patients with advanced squamous cell carcinoma of the oral cavity (pT4a).

Features	HR	95% IC	*p*
Perineural invasion	0.985	0.506–1.916	0.964
Angiolymphatic invasion	1.325	0.824–2.129	0.245
Depth of invasion > 10 mm	1.749	0.823–3.716	0.146
Lymph node metastases	a	a	a
Extracapsular spread	1.697	1.018–2.831	0.043
Hb < 10 g/dL	0.994	0.530–1.865	0.986
RDW > 14.3%	2.210	1.378–3.551	0.001
Neutrophil/lymphocyte ratio > 2.2	0.972	0.492–1.917	0.936
Percentage of loss compared to usual weight > 10%	1.679	1.046–2.693	0.032

HR, Hazard Ratio; 95% CI, Confidence Interval 95%; *p*, value of *p*.

a Reduced degree of freedom due to linearly dependent or constant variables.

The graphical demonstration of overall survival shows that patients who lost more than 10% of their weight had a median survival of 13 months compared to 49 months among those who lost less than 10%. For individuals with RDW values greater than 14.3%, they had a median survival of 11 months compared to 42 months for those who had the lowest values. Finally, patients who had extracapsular spread reached a median survival of 10 months compared to 26 months for those who did not have this condition. Data from the survival analysis and Kaplan-Meier curves are shown, respectively, in Table 5 and Fig. 1.

**Table 5:** Survival analysis with independent risk variables for general death in patients with advanced oral squamous cell carcinoma (pT4a).

	Events	Cumulative survival	Median survival
**Extracapsular spread**			
Absent	23/41	38.5%	26 months
Present	55/66	6.6%	10 months
**RDW**			
≤14.3%	62/123	31.1%	42 months
>14.3%	38/54	27.5%	11 months
**Percentage of loss in relation to usual weight**			
≤10%	60/124	37.0%	49 months
>10%	37/49	8.9%	13 months

## Discussion

The percent of weight loss and high rates of RDW were independent predictors of death in patients with advanced OSCC. This is a relevant finding for this specific population because they are potentially modifiable factors, even in the preoperative period, and can lead to better outcomes after surgery.

Some studies have reported an association between high levels of RDW and increased mortality in the general population.^25^, ^26^ High levels of RDW are believed to be caused by chronic inflammation and poor nutritional status (for example, deficiency of iron, folate and vitamin B12).^27^ There are published clinical studies on the interaction between RDW and malignant tumors.^28^, ^29^ The study by Koma et al.^27^ on lung cancer concluded that cases with the highest levels of RDW are associated with lower survival, as RDW is associated with several factors that reflect the inflammation and malnutrition state of patients with lung cancer, and this index can be used as a new marker to determine a patient's general condition. A meta-analyses by Hu et al.^30^ including 16 studies on various cancer locations concluded that elevated RDW was an unfavorable predictor of prognosis.

In OSCC there are few published studies on the subject. Ge et al. reported on 236 patients with oral squamous cell carcinoma and showed that a high RDW was connected with poor overall survival^31^ and Miszczyk et al. compared 74 patients with tongue tumors treated with chemoradiation and found that OS was significantly lower in patients with RDW ≥ 13.5% compared with patients with RDW < 13.5% (67% vs. 26%).^32^ However, Tangthongkum et al. reported no significant differences in OS between the high and low RDW groups in a retrospective study on 374 patients with oral cancer,^33^ so there is still no consensus on the subject.^33^ In our study, we observed that 54 patients with high RDW had a median survival of 11 months, while patients with low RDW took 42 months to reach it. These data corroborate what was previously found for other malignancies and now in a cohort of advanced OSCC (pT4a).

The percentage of weight lost prior to treatment has also been associated with shorter survival in various cancers. In esophageal cancer patients treated with surgery and chemotherapy, Yu et al.^34^ demonstrated that patients with pre-treatment weight lost higher than 5% had worse OS in three years than those that did not (HR = 1.89, 95% CI 1.07–3.32). In head and neck cancer, Orell-Kotikangas et al.^35^ showed that the presence of cachexia was associated with lower OS and disease-free survival, five-year OS decreased from 69% in cachectic patients to 30% in non-cachectic patients. In the present series, weight loss higher than 10% was identified as an independent risk factor for death.

These findings are of great importance since measures can be established to try to mitigate the weight loss. Due to the characteristics of OSCC in advanced stages, with impaired chewing and swallowing capacity, preoperative use of enteral nutrition that provides greater caloric intake should be carefully considered.^36^ Such therapy today is based on a hyperproteic, hypercaloric diet associated with Omega 3 to stabilize weight loss and add anti-inflammatory factors for recovery after scheduled surgical treatment.^37^ Alternatively, the consistency of the diet can be changed to allow oral feeding. However, many patients still need hybrid nutritional regimens, as demonstrated in our study that after one year of treatment, 43 patients continued to use exclusive enteral therapy, and 171 (96.1%) used a feeding tube at some point during treatment. In the approach to nutrition, especially in surgical cases, care should aim to adjust the caloric needs of the individual, especially in groups with marked weight loss, and early care is important from the nutritional and multidisciplinary teams (psychology, nursing, social assistance, speech therapy).^38^

The presence of lymph node metastasis is the main determinant of a worse prognosis in head and neck cancer patients.^4^ In OSCC, this scenario is the same and the prevalence of regional disease is approximately 50%.^39^ Extracapsular spread forecasts an even worse prognosis in these patients^40^ and is absolutely frequent specially in individuals with advanced disease. We also found this characteristic as an independent factor of worse survival in our cohort of pT4a patients.

The present study has some limitations. Due to its retrospective nature, it was not possible to obtain all the necessary data at the same moment for all patients. Furthermore, working with data that require the patient’s assistance, such as their usual weight, can lead to inherent information bias. Furthermore, the study only estimated the risk of dying due to OSCC; we were unable to develop a nutritional protocol that could be applied to all patients to obtain a more reliable result with more individualized monitoring of patients. The institution has a nutritional protocol for surgical patients, but it has not been uniformly applied to all patients.

## Conclusions

Patients with advanced squamous cell carcinoma of the oral cavity presented significant weight loss and immune compromise. Increased values of RDW and higher percentages of weight loss in relation to the individual's usual weight, together with extracapsular spread of lymph node metastases, were risk factors for lower survival, regardless of other known clinical and anatomopathological characteristics. Patients undergoing surgery and adjuvant treatments must receive a complete nutritional evaluation, adequate nutritional guidance and, if necessary, the use of enteral nutritional therapy. Nutritional intervention can be effective, preventing nutritional deterioration, which can improve the clinical outcomes of these patients.^41^, ^42^

## Authors’ contributions

Both authors equally contributed to this study.

LFMT: data acquisition, data analysis, manuscript critical review.

IFK: data acquisition, data analysis, manuscript preparation.

AKNL: data analysis, manuscript preparation.

MAVK: study design, manuscript preparation.

GASL: data acquisition, manuscript critical review.

RAD: study design, manuscript critical review.

LPK: study design, manuscript critical review.

LLM: study design, data analysis, manuscript preparation.

All authors have read and approved the final version of the manuscript.

## Funding

Public São Paulo State Research Support Foundation (Fundação de Amparo à Pesquisa do Estado de São Paulo – FAPESP – grant number 2016/01740-6) and National Brazilian Government Research Agency (Conselho Nacional de Desenvolvimento Científico e Tecnológico – CNPq – grant number 800605/2018-7).

## Conflicts of interest

All authors have read and approved the final manuscript and do not have actual, potential, or apparent conflict of interest with regard to the manuscript submitted for review.

## References

[1] Rogers S.N., Ahad S.A., Murphy A.P. (2007). A structured review and theme analysis of papers published on’ quality of life’ in head and neck cancer: 2000-2005. Oral Oncol..

[2] Matos L.L., Miranda G.A., Cernea C.R. (2015). Prevalence of oral and oropharyngeal human papillomavirus infection in Brazilian population studies: a systematic review. Braz J Otorhinolaryngol..

[3] WHO (2000).

[4] d’Alessandro A.F., Pinto F.R., Lin C.S., Kulcsar M.A., Cernea C.R., Brandao L.G. (2015). Oral cavity squamous cell carcinoma: factors related to occult lymph node metastasis. Braz J Otorhinolaryngol..

[5] INCA (2019).

[6] Mantovani A., Allavena P., Sica A., Balkwill F. (2008). Cancer-related inflammation. Nature..

[7] Oliveira F.P., Santos A., Viana M.S., Alves J.L., Pinho N.B., Reis P.F. (2015). Perfil nutricional de pacientes com câncer de cavidade oral em pré-tratamento antineoplásico. Rev Bras Cancerol..

[8] Pinto F.R., Matos L.L., Gumz Segundo W., Vanni C.M., Rosa D.S., Kanda J.L. (2011). Tobacco and alcohol use after head and neck cancer treatment: influence of the type of oncological treatment employed. Rev Assoc Med Bras (1992)..

[9] Gaudet M.M., Olshan A.F., Chuang S.C., Berthiller J., Zhang Z.F., Lissowska J. (2010). Body mass index and risk of head and neck cancer in a pooled analysis of case-control studies in the International Head and Neck Cancer Epidemiology (INHANCE) Consortium. Int J Epidemiol..

[10] Kumar S., Mahmud N., Goldberg D.S., Datta J., Kaplan D.E. (2021). Disentangling the obesity paradox in upper gastrointestinal cancers: weight loss matters more than body mass index. Cancer Epidemiol..

[11] Liu S.A., Tsai W.C., Wong Y.K., Lin J.C., Poon C.K., Chao S.Y. (2006). Nutritional factors and survival of patients with oral cancer. Head Neck..

[12] Karnell L.H., Sperry S.M., Anderson C.M., Pagedar N.A. (2016). Influence of body composition on survival in patients with head and neck cancer. Head Neck..

[13] Nasser H., St John M. (2018). Immunotherapeutic approaches to head and neck cancer. Crit Rev Oncog..

[14] Kwon H.C., Kim S.H., Oh S.Y., Lee S., Lee J.H., Choi H.J. (2012). Clinical significance of preoperative neutrophil-lymphocyte versus platelet-lymphocyte ratio in patients with operable colorectal cancer. Biomarkers..

[15] Rassouli A., Saliba J., Castano R., Hier M., Zeitouni A.G. (2015). Systemic inflammatory markers as independent prognosticators of head and neck squamous cell carcinoma. Head Neck..

[16] Sharaiha R.Z., Halazun K.J., Mirza F., Port J.L., Lee P.C., Neugut A.I. (2011). Elevated preoperative neutrophil: lymphocyte ratio as a predictor of postoperative disease recurrence in esophageal cancer. Ann Surg Oncol..

[17] Lippi G., Targher G., Montagnana M., Salvagno G.L., Zoppini G., Guidi G.C. (2009). Relation between red blood cell distribution width and inflammatory biomarkers in a large cohort of unselected outpatients. Arch Pathol Lab Med..

[18] Celik A., Aydin N., Ozcirpici B., Saricicek E., Sezen H., Okumus M. (2013). Elevated red blood cell distribution width and inflammation in printing workers. Med Sci Monit..

[19] Li Z., Hong N., Robertson M., Wang C., Jiang G. (2017). Preoperative red cell distribution width and neutrophil-to-lymphocyte ratio predict survival in patients with epithelial ovarian cancer. Sci Rep..

[20] Bozkurt G., Korkut A.Y., Soytaş P., Dizdar S.K., Erol Z.N. (2019). The role of red cell distribution width in the locoregional recurrence of laryngeal cancer. Braz J Otorhinolaryngol..

[21] Silva M.P.N. (2006). Síndrome da anorexia-caquexia em portadores de câncer. Rev Bras Cancerol..

[22] Alshadwi A., Nadershah M., Carlson E.R., Young L.S., Burke P.A., Daley B.J. (2013). Nutritional considerations for head and neck cancer patients: a review of the literature. J Oral Maxillofac Surg..

[23] Amin M.B., Edge S.B., Greene F.L., Byrd D.R., Brookland R.K., Washington M.K. (2017).

[24] OPAS (2002). Organización Panamericana de la Salud. División de Promoción y Protección de la Salud (HPP). Encuesta Multicentrica salud beinestar y envejecimiento (SABE) em América Latina el Caribe: Informe Preliminar Kingston. http://www.opas.org/program/sabe.htm.

[25] Patel K.V., Ferrucci L., Ershler W.B., Longo D.L., Guralnik J.M. (2009). Red blood cell distribution width and the risk of death in middle-aged and older adults. Arch Intern Med..

[26] Patel K.V., Semba R.D., Ferrucci L., Newman A.B., Fried L.P., Wallace R.B. (2010). Red cell distribution width and mortality in older adults: a meta-analysis. J Gerontol A Biol Sci Med Sci..

[27] Koma Y., Onishi A., Matsuoka H., Oda N., Yokota N., Matsumoto Y. (2013). Increased red blood cell distribution width associates with cancer stage and prognosis in patients with lung cancer. PLoS One..

[28] Langius J.A., van Dijk A.M., Doornaert P., Kruizenga H.M., Langendijk J.A., Leemans C.R. (2013). More than 10% weight loss in head and neck cancer patients during radiotherapy is independently associated with deterioration in quality of life. Nutr Cancer..

[29] Seretis C., Seretis F., Lagoudianakis E., Gemenetzis G., Salemis N.S. (2013). Is red cell distribution width a novel biomarker of breast cancer activity? Data from a pilot study. J Clin Med Res..

[30] Hu L., Li M., Ding Y., Pu L., Liu J., Xie J. (2017). Prognostic value of RDW in cancers: a systematic review and meta-analysis. Oncotarget..

[31] Ge W., Xie J., Chang L. (2018). Elevated red blood cell distribution width predicts poor prognosis in patients with oral squamous cell carcinoma. Cancer Manag Res..

[32] Miszczyk M., Jabłońska I., Magrowski Ł., Masri O., Rajwa P. (2020). The association between RDW and survival of patients with squamous cell carcinoma of the tongue. Simple, cheap and convenient?. Rep Pract Oncol Radiother..

[33] Tangthongkum M., Tiyanuchit S., Kirtsreesakul V., Supanimitjaroenporn P., Sinkitjaroenchai W. (2017). Platelet to lymphocyte ratio and red cell distribution width as prognostic factors for survival and recurrence in patients with oral cancer. Eur Arch Otorhinolaryngol..

[34] Yu X.L., Yang J., Chen T., Liu Y.M., Xue W.P., Wang M.H. (2018). Excessive pretreatment weight loss is a risk factor for the survival outcome of esophageal carcinoma patients undergoing radical surgery and postoperative adjuvant chemotherapy. Can J Gastroenterol Hepatol..

[35] Orell-Kotikangas H., Österlund P., Mäkitie O., Saarilahti K., Ravasco P., Schwab U. (2017). Cachexia at diagnosis is associated with poor survival in head and neck cancer patients. Acta Otolaryngol..

[36] Ackerman D., Laszlo M., Provisor A., Yu A. (2018). Nutrition management for the head and neck cancer patient. Cancer Treat Res..

[37] Di Renzo L., Marchetti M., Cioccoloni G., Gratteri S., Capria G., Romano L. (2019). Role of phase angle in the evaluation of effect of an immuno-enhanced formula in post-surgical cancer patients: a randomized clinical trial. Eur Rev Med Pharmacol Sci..

[38] Shellenberger T.D., Weber R.S. (2018). Multidisciplinary team planning for patients with head and neck cancer. Oral Maxillofac Surg Clin North Am..

[39] Pinto F.R., de Matos L.L., Palermo F.C., Kulcsar M.A., Cavalheiro B.G., de Mello E.S. (2014). Tumor thickness as an independent risk factor of early recurrence in oral cavity squamous cell carcinoma. Eur Arch Otorhinolaryngol..

[40] Mermod M., Tolstonog G., Simon C., Monnier Y. (2016). Extracapsular spread in head and neck squamous cell carcinoma: a systematic review and meta-analysis. Oral Oncol..

[41] Corry J., Poon W., McPhee N., Milner A.D., Cruickshank D., Porceddu S.V. (2008). Randomized study of percutaneous endoscopic gastrostomy versus nasogastric tubes for enteral feeding in head and neck cancer patients treated with (chemo)radiation. J Med Imaging Radiat Oncol..

[42] Isenring E.A., Bauer J.D., Capra S. (2007). Nutrition support using the American Dietetic Association medical nutrition therapy protocol for radiation oncology patients improves dietary intake compared with standard practice. J Am Diet Assoc..

